# Spirometric Lung Functions in Type 2 Diabetes Mellitus: A Hospital-Based Study

**DOI:** 10.7759/cureus.38919

**Published:** 2023-05-12

**Authors:** Ashish Sharma, Anupriya Sharma, Rakesh Chauhan

**Affiliations:** 1 Neurology, All India Institute of Medical Sciences, Bilaspur, IND; 2 Dentistry, Dr. Radhakrishnan Government Medical College, Hamirpur, IND; 3 Internal Medicine, Dr. Radhakrishnan Government Medical College, Hamirpur, IND

**Keywords:** hba1c, pulmonary function test, spirometry, lungs, diabetes mellitus

## Abstract

Objective

This cross-sectional case-control study was conducted with the aim to analyze spirometric lung functions in type 2 diabetes mellitus (T2DM) patients and to correlate the spirometric dysfunction with (a) duration of diabetes, b) metabolic control of diabetes, and c) microvascular complications of diabetes.

Methods

Pulmonary function tests (PFTs) were performed in 50 T2DM patients and 50 normal healthy controls aged <80 years by using an electronic spirometer. The PFTs recorded were as follows: forced vital capacity (FVC), forced expiratory volume in one second (FEV1), FEV1%, forced expiratory flow 25 (FEF25), forced expiratory flow 25-75 (FEF25-75), and peak expiratory flow rate (PEFR). The glycated hemoglobin (HbA1c) of all the patients was measured by affinity chromatography using the NycoCard HbA1C kit. The assessment of diabetic microvascular complications was performed as follows: peripheral neuropathy was done using Michigan Neuropathy Screening Instrument (MNSI), diabetic retinopathy using fundus examination, and diabetic nephropathy using solid phase/sandwich-format/immunometric assay using NycoCard U-albumin kit. PFTs of diabetic patients and controls were compared by applying an independent sample t-test. The correlation between FVC and FEV1, and HbA1c and duration of illness in diabetic patients was analyzed by applying the Pearson coefficient.

Results

The cases had low FVC (103.82 ±24.43 vs. 116.08 ±13.66), FEV1 (101.36 ±24.23 vs. 110.26 ±14.39), FEV1% (97.56 ±8.64 vs. 103.84 ±5.06), PEFR (101.52 ±27.18 vs. 116.96 ±14.96), and FEF 25-75 (73.56 ±29.19 vs. 98.40 ±14.45) compared to controls, and the difference was statistically significant. A significant negative correlation was found between spirometry parameters and duration of illness as well as HbA1c. Spirometric lung dysfunction also negatively correlated with microvascular complications of diabetes. Among various microvascular complications, retinopathy correlated best with various spirometric parameters.

Conclusion

Based on our findings, T2DM patients had a significant decrease in their spirometric indices. The pattern of spirometric dysfunction was suggestive of "mixed ventilatory dysfunction". The study results highlight the need to include PFTs in the periodic check-up as part of the comprehensive management of diabetic patients. Hence, pulmonary function should be included in the periodic comprehensive diabetic check for the holistic management of these patients.

## Introduction

Diabetes is a chronic systemic metabolic disorder that affects 463 million people over the age of 20 years worldwide and is expected to affect more than 570 million by 2030 [1]. Diabetes is a global health problem and is a leading cause of cardiovascular disease, kidney failure, blindness, and lower limb amputation [2]. Globally, it is reported that 4.2 million deaths were attributed to type 2 diabetes mellitus (T2DM) and its complications in 2019 [1]. T2DM affects all organs in the human body. It develops relatively slowly, and various studies have found fibrotic changes in the lungs [3] and pulmonary microcirculation disorders in diabetic patients [4]. There have been concerted attempts to investigate abnormal respiratory conditions in general diabetic patients [5,6].

Although interest in this condition has increased in recent years, the findings of various studies show high variability. A meta-analysis by van den Borst et al. reported data about diffusing capacity of the lungs for carbon monoxide and concluded that T2DM is associated with impaired pulmonary function in a restrictive pattern [7]. A recent systemic review and meta-analysis by Saini et al. suggest that pulmonary pathology is more prevalent among patients with T2DM than those without, with an increased risk of chronic obstructive pulmonary disease (COPD), pulmonary fibrosis, and other chronic lung diseases [8]. Sufficient attention is not given in our country to diabetes patients with impaired lung functions. The existing literature regarding mechanical abnormalities in lung function is inconsistent and equivocal. Pulmonary function tests (PFTs) among T2DM patients have shown varied results with frequently contradicting findings; some studies indicate a reduction in spirometric parameters, whereas others have demonstrated no change compared with non-diabetics [9,10]. The impaired forced vital capacity (FVC) and forced expiratory volume in one second (FEV1) have been reported as emerging novel risk factors for T2DM [11]. In the context of these reports, the spirometric parameters have gained more popularity. However, spirometry is still not used routinely as part of the management system in diabetic patients. The role of spirometry is neither fully explored nor fully utilized to achieve the desired quality of life in terms of managing diabetes mellitus. The inclusion of spirometry in the algorithm of the routine assessment of diabetes may serve as a brief reference material for diabetes management teams.

Therefore, the aim of the present study was to study the spirometric lung functions in T2DM patients and to correlate the spirometric dysfunction with the duration of diabetes, metabolic control of diabetes, and microvascular complications of diabetes.

## Materials and methods

We employed a cross-sectional case-control design for this study. A case was defined as a previously diagnosed T2DM patient attending the Diabetic Clinic at the Medical College. The controls were age-matched healthy volunteers who were spouses of the cases enrolled in the study. Cases were matched with controls in terms of age, sex, and height. Informed written consent was obtained from all subjects willing to take part in the study.

The sample size was calculated by assuming the prevalence of restrictive lung disease in the diabetic population to be 27-30% because many previous studies have shown this estimate to be around 30% [12]. Considering a 95% confidence level and an absolute precision of 10%, the sample size of 96 was calculated.

The simple formula used for calculating the adequate sample size in this study was 𝑛=𝑍2p(1−p)/𝑑2. A total of 50 cases and an equal number of controls were included in the study. Subjects were randomly selected using the systematic random sampling technique. Details were recorded as per the predesigned proforma. The subjects with the following characteristics were excluded from the study: smokers; aged more than 85 years; body mass index (BMI) of more than or equal to 30; historic, clinical, or radiological evidence of respiratory illness; historic, clinical, or electrocardiographic evidence of cardiac illness; historic or clinical evidence of neuromuscular illness likely to affect the performance of spirometric PFTs; limited joint mobility as assessed by prayer sign; inability to perform acceptable and reproducible spirometry; and unwillingness to participate in the study.

After recording demographic details, the cases were screened for exclusion criteria: by history, clinical examination, and investigations (CXR/ECG). History included smoking history, age, history of previous hospital admissions, history of cardiorespiratory symptoms, and duration of diabetes. Duration of diabetes was defined as the period between age at diagnosis and age when examined for the study. In the examination, emphasis was given to the measurement of BMI, a detailed examination of the cardiorespiratory system, and an assessment of limited joint mobility by prayer sign. Assessment for peripheral neuropathy was done by using Michigan Neuropathy Screening Instrument (MNSI; clinical portion). A score >2/8 was considered significant. Fundus examination for diabetic retinopathy was done by an experienced ophthalmologist. At least one microaneurysm in one eye or worse as assessed by direct or indirect ophthalmoscopy through dilated pupils was considered significant for diabetic retinopathy. Nephropathy was defined as microalbuminuria or worse. The method of measurement was solid phase/sandwich-format /immunometric assay using the NycoCard U-albumin kit. Any random urine sample was taken as suitable for measuring microalbuminuria. The measuring range of the kit was 5-200 mg/L with a measuring interval of 1 mg/L; 95% sensitivity and 93% specificity have been found as compared to routine laboratory methods like radioimmunoassay techniques, immunoturbidometric assay, and nephelometry. Albuminuria of <20 mg/L was taken as normoalbuminuria, 20-200 mg/L as microalbuminuria, and >200 mg/L as macroalbuminuria. Microalbuminuria was considered valid only if the following were excluded: exercise within 24 hours, infection, fever, congestive heart failure, marked hyperglycemia, marked hypertension, pyuria, and hematuria. For the purpose of the present study, microvascular complications were considered as a binary variable (present/absent).

Glycated hemoglobin (HbA1C) was estimated to measure the metabolic control of diabetes in patients. Estimation of HbA1C was done in venous blood with EDTA by affinity chromatography [13] using a NycoCard HbA1C kit. The assay has a measuring range of 3-18% HbA1C, and measuring interval of 0.1% HbA1C, and a reference range of 4.5-6.3%. The coefficient of variation was below 5% in the whole measuring range.

After recording demographic details, the controls were screened for exclusion criteria. Glycemic status was confirmed by measuring fasting blood sugar (FBS). Blood glucose estimation was done in fasting serum by enzymatic GOD-POD method by the endpoint colorimetry using semi-auto analyzer ERBA Chem 5 Plus.

Spirometric PFT was done after recording all baseline parameters. Spirometric PFTs were performed by a trained technician under direct supervision by using an electronic spirometer (Vitalograph Compact; Vitalograph Ltd., Buckingham, England), which fulfilled equipment performance criteria and equipment validation as per the American Thoracic Society (ATS) guidelines [14]. It is a portable spirometer with a Fleisch pneumotachograph type of flow head. The procedure was performed according to the acceptability and reproducibility criteria of ATS, after giving detailed instructions to the subjects in their own language. Each subject provided at least three acceptable tracings, from which FVC, FEV1, FEV1/FVC ratio, peak expiratory flow rate (PEFR), and forced expiratory flow 25-75 (FEF 25-75) were measured. The largest values among the minimum three acceptable maneuvers were used for analysis. All values were corrected for body temperature, air pressure, and water saturation. Spirometric data was recorded as follows: (a) absolute measures and (b) percentage of those predicted for age, sex, and height.

Statistical analysis

Data were presented as mean ±standard deviation (SD), proportion, and frequency. Statistical significance of the observed difference between spirometric measures of cases as compared to controls was assessed by independent sample t-test. A p-value <0.05 was considered statistically significant. The association of duration, metabolic control, and microvascular complications of DM with lung functions was assessed by one-way ANOVA and independent sample t-test. A p-value <0.05 was considered statistically significant. Pearson correlation coefficient was used to compare the strength of correlation among diabetic factors and microvascular complications of diabetes with spirometric parameters. Statistical analysis was performed using the IBM SPSS Statistics software (IBM Corp., Armonk, NY).

## Results

A total of 50 cases (T2DM patients) and an equal number of age-, sex-, and height-matched controls were evaluated. The age of the patients ranged from 35 to 78 years. The mean age was 56.42 years with an SD of 10.63 years (Table 1).

**Table 1:** Age of the patients

Age group, years	Frequency	Percent
31-40	3	6
41-50	13	26
51-60	16	32
61-70	13	26
>70	5	10
Total	50	100

Table 2 shows the gender-wise distribution of the patients.

**Table 2:** Gender-wise distribution

Gender	Frequency	Percent
Male	28	56
Female	22	44
Total	50	100

Table 3 shows the distribution of patients as per the duration of diabetes; 42% of the patients had a duration of diabetes of less than 10 years, and 58% had diabetes for more than 10 years. The mean duration of diabetes was 14.8 years with an SD of 5.8 years.

**Table 3:** Distribution of patients by the duration of diabetes

Duration of diabetes, years	Frequency	Percent
0-5	9	18
6-10	12	24
11-15	21	42
>15	8	16
Total	50	100

Table 4 shows the glycemic control (HbA1C) in patients; 32% of patients had good glycemic control whereas 68% of patients had poor glycemic control. The mean level of HbA1C was 8.8 with an SD of 2.4. The majority of patients had an HbA1C level between 7-10% (48%); 20% of patients had an HbA1C level >10%.

**Table 4:** Glycemic control (HbA1C)

HbA1C, %	Frequency	Percent
6-7	16	32
7-10	24	48
>10	10	20
Total	50	100

Tables 5-7 show the frequency of complications: (i) peripheral neuropathy, (ii) retinopathy, and iii) nephropathy.

**Table 5:** Peripheral neuropathy

Neuropathy	Frequency	Percent
Absent	28	56
Present	22	44
Total	50	100

**Table 6:** Retinopathy

Retinopathy	Frequency	Percent
Absent	26	52
Present	24	48
Total	50	100

**Table 7:** Nephropathy

Nephropathy	Frequency	Percent
Absent	30	60
Present	20	40
Total	50	100

Table 8 shows the distribution of various complications; 64% of the patients had microvascular complications of diabetes in some form - single complication (9): retinopathy (6), neuropathy (2), and nephropathy (1); two complications (11): neuropathy + nephropathy (5), neuropathy + retinopathy (4), and retinopathy + nephropathy (2).

**Table 8:** Distribution of various complications

Number of complications	Frequency	Percent
No complication	18	36
Single complication	9	18
Two complications	11	22
Three complications	12	24
Total	50	100

Table 9 shows spirometric parameters in cases and controls; there was a significant difference (decrease) in all the spirometric measures among cases. The average difference was 13.54%. FEF25-75 showed the highest difference followed by PEFR, FVC, FEV1, and FEV1%.

**Table 9:** Spirometric parameters in cases and controls P-value <0.05 was considered statistically significant SD: standard deviation; FVC: forced vital capacity; FEV1: forced expiratory volume in one second; PEFR: peak expiratory flow rate; FEF: forced expiratory flow

Spirometric parameters	Cases, mean ±SD	Control, mean ±SD	Difference, mean	P-value (paired sample t-test)
FVC	103.82 ±24.43	116.08 ±13.66	12.26	<0.001
FEV1	101.36 ±24.23	110.26 ±14.39	8.90	<0.001
FEV1%	97.56 ±8.64	103.84 ±5.06	6.28	<0.001
PEFR	101.52 ±27.18	116.96 ±14.96	15.44	<0.001
FEF25-75	73.56 ±29.19	98.40 ±14.45	24.84	<0.001

Tables 10-11 show the comparison of spirometric parameters with various diabetic-related factors. (I) Duration of diabetes: it was significantly correlated with FEF25-75, FEV1, PEFR, and FVC in descending order. However, It was not significantly correlated with FEV1% (Table 10). (ii) Glycemic control (HbA1C): it was significantly correlated with FEF25-75, PEFR, FEV1, FVC, and FEV1% in descending order (Table 11).

**Table 10:** Comparison of spirometric parameters with the duration of diabetes P-value <0.05 was considered statistically significant ANOVA: analysis of variance; SD: standard deviation; FVC: forced vital capacity; FEV1: forced expiratory volume in one second; PEFR: peak expiratory flow rate; FEF: forced expiratory flow

Spirometric parameters, mean ±SD	Duration of diabetes in years (number of patients)				P-value (ANOVA)
	0-5 (9)	6-10 (12)	11-15 (21)	>15 (8)	
FVC	122.11 ±20.78	104.41 ±23.46	100.52 ±25.59	91 ±17.27	0.049
FEV1	121.77 ±23.39	104.58 ±21.05	97.38 ±23.27	84 ±17.21	0.007
FEV1%	99.33 ±8.91	100.25 ±9.39	97.28 ±7.11	92.25 ±10.02	0.207
PEFR	120.22 ±33.04	109.50 ±20.52	93.04 ±20.80	90.75 ±33.45	0.030
FEF25-75	92 ±35.05	89.33 ±29.02	65.66 ±20.25	49.87 ±19.31	0.001

**Table 11:** Comparison of spirometric parameters with glycemic control (HbA1C) P-value <0.05 was considered statistically significant ANOVA: analysis of variance; SD: standard deviation; FVC: forced vital capacity; FEV1: forced expiratory volume in one second; PEFR: peak expiratory flow rate; FEF: forced expiratory flow

Spirometric parameters, mean ±SD	HbA1C in % (number of patients)			P-value (ANOVA)
	6-7 (16)	7-10 (24)	>10 (10)	
FVC	116.56 ±28.07	103.25 ±20.18	84 ±13.97	0.004
FEV1	117.87 ±25.04	99.91 ±18.11	78.40 ±15.43	0.000
FEV1%	101.50 ±9.30	97.20 ±6.81	92.10 ±9.12	0.022
PEFR	117.50 ±21.93	93.53 ±20.76	84.50 ±36.18	0.005
FEF25-75	99.31 ±26.66	67.29 ±19.82	47.40 ±20.14	0.000

Tables 12-15 show the comparison of spirometric parameters with microvascular complications of diabetes. (i) Neuropathy: the presence of neuropathy correlated significantly with PEFR, FEF25-75, and FEV1 in descending order. However, It did not correlate significantly with FVC and FEV1% (Table 12). (ii) Retinopathy: the presence of retinopathy correlated significantly with FEF25-75, FEV1, PEFR, and FVC in descending order. However, It did not correlate significantly with FEV1% (Table 13). (iii) Nephropathy: the presence of nephropathy correlated significantly with PEFR, FEF25-75, and FEV1 in descending order. However, It did not correlate significantly with FVC and FEV1% (Table 14). (iv) Number of complications: the increase in the number of complications correlated significantly with PEFR, FEV1, FVC, and FEF25-75 in descending order. However, It did not correlate significantly with FEV1% (Table 15).

**Table 12:** Comparison of spirometric parameters with neuropathy P-value <0.05 was considered statistically significant SD: standard deviation; FVC: forced vital capacity; FEV1: forced expiratory volume in one second; PEFR: peak expiratory flow rate; FEF: forced expiratory flow

Spirometric parameters, mean ±SD	Neuropathy (number of patients)		P-value (independent sample t-test)
	Present (22)	Absent (28)	
FVC	97.13 ±22.70	109.07 ±24.85	0.087
FEV1	93.72 ±21.48	107.35 ±24.94	0.047
FEV1%	96.63 ±10.16	98.28 ±7.35	0.509
PEFR	87.40 ±24.84	112.60 ±23.89	0.001
FEF25-75	64.13 ±25.79	80.96 ±29.99	0.042

**Table 13:** Comparison of spirometric parameters with retinopathy P-value <0.05 was considered statistically significant SD: standard deviation; FVC: forced vital capacity; FEV1: forced expiratory volume in one second; PEFR: peak expiratory flow rate; FEF: forced expiratory flow

Spirometric parameters, mean ±SD	Retinopathy (number of patients)		P-value (independent sample t-test)
	Present (24)	Absent (26)	
FVC	95.54 ±21.67	111.46 ±24.73	0.02
FEV1	90.83 ±19.61	111.07 ±24.35	0.002
FEV1%	95.58 ±10.25	99.38 ±6.53	0.122
PEFR	91.04 ±24.89	111.19 ±25.98	0.007
FEF25-75	60.66 ±24.36	85.46 ±28.58	0.002

**Table 14:** Comparison of spirometric parameters with nephropathy P-value <0.05 was considered statistically significant SD: standard deviation; FVC: forced vital capacity; FEV1: forced expiratory volume in one second; PEFR: peak expiratory flow rate; FEF: forced expiratory flow

Spirometric parameters, mean ±SD	Nephropathy (number of patients)		P-value (independent sample t-test)
	Present (20)	Absent (30)	
FVC	96.15 ±23.64	108.93 ±23.98	0.069
FEV1	92.45 ±22.53	107.30 ±23.84	0.032
FEV1%	96.40 ±10.41	98.33 ±7.33	0.444
PEFR	87.50 ±25.40	110.86 ±24.48	0.002
FEF25-75	62 ±23.67	81.26 ±30.31	0.021

**Table 15:** Comparison of spirometric parameters with the number of complications P-value <0.05 was considered statistically significant ANOVA: analysis of variance; SD: standard deviation; FVC: forced vital capacity; FEV1: forced expiratory volume in one second; PEFR: peak expiratory flow rate; FEF: forced expiratory flow

Spirometric parameters, mean ±SD	Number of complications (number of patients)				P-value (ANOVA)
	Nil (18)	Single (9)	Two (11)	Three (12)	
FVC	110.88 ±25.88	104.55 ±24.18	114.18 ±20.53	83.16 ±12.69	0.004
FEV1	111.22 ±26.70	102.44 ±21.46	107.09 ±17.29	80.50 ±15.49	0.003
FEV1%	99.72 ±6.89	98.66 ±7.51	94.09 ±6.44	96.66 ±12.62	0.375
PEFR	116.44 ±26.32	105.77 ±19.07	97.45 ±21.11	79.66 ±25.16	0.002
FEF25-75	88.83 ±31.32	75.44 ±26.83	65.72 ±16.94	56.41 ±26.97	0.015

Table 16 shows the comparison of the degree of correlation between spirometric parameters and the duration and metabolic control of diabetes. Glycemic control correlated comparatively more strongly with all spirometric parameters than the duration of diabetes.

**Table 16:** Comparison of the degree of correlation between spirometric parameters and the duration and metabolic control of diabetes P-value <0.05 was considered statistically significant FVC: forced vital capacity; FEV1: forced expiratory volume in one second; PEFR: peak expiratory flow rate; FEF: forced expiratory flow

Spirometric parameters		Duration of diabetes	HbA1C
FVC	Pearson correlation	-0.377	-0.403
	Sig. (2-tailed)	0.007	0.004
FEV1	Pearson correlation	-0.472	-0.509
	Sig. (2-tailed)	0.001	0.000
FEV1%	Pearson correlation	-0.259	-0.354
	Sig. (2-tailed)	0.069	0.012
PEFR	Pearson correlation	-0.400	-0.559
	Sig. (2-tailed)	0.004	0.000
FEF25-75	Pearson correlation	-0.511	-0.526
	Sig. (2-tailed)	0.000	0.000

Table 17 shows the comparison of the strength of correlation among microvascular complications of diabetes with spirometric parameters: retinopathy, nephropathy, and neuropathy correlated in descending order with all spirometric parameters (e.g., FVC, FEV1, FEV1%, and FEF25-75) except for PEFR. Neuropathy, nephropathy, and retinopathy correlated with PEFR in descending order. The number of complications had the highest strength of correlation with all spirometric parameters except for FEV1%, which was second to retinopathy.

**Table 17:** Comparison of the strength of correlation among microvascular complications of diabetes with spirometric parameters P-value <0.05 was considered statistically significant FVC: forced vital capacity; FEV1: forced expiratory volume in one second; PEFR: peak expiratory flow rate; FEF: forced expiratory flow

Spirometric parameters		Neuropathy	Retinopathy	Nephropathy	Number of complications
FVC	Pearson correlation	-0.245	-0.329	-0.259	-0.353
	Sig. (2-tailed)	0.087	0.020	0.069	0.012
FEV1	Pearson correlation	-0.282	-0.421	-0.303	-0.430
	Sig. (2-tailed)	0.047	0.002	0.032	0.002
FEV1%	Pearson correlation	-0.096	-0.222	-0.111	-0.193
	Sig. (2-tailed)	0.509	0.122	0.444	0.180
PEFR	Pearson correlation	-0.465	-0.374	-0.425	-0.522
	Sig. (2-tailed)	0.001	0.007	0.002	0.000
FEF25-75	Pearson correlation	-0.289	-0.429	-0.327	-0.448
	Sig. (2-tailed)	0.042	0.002	0.021	0.001

Table 18 shows the profile of glycemic control in various subgroups divided on the basis of duration of diabetes/profile of the duration of diabetes in various subgroups divided on the basis of glycemic control.

**Table 18:** Profile of glycemic control in various subgroups divided on the basis of duration of diabetes/profile of the duration of diabetes in various subgroups divided on the basis of glycemic control

Level of HBA1C in % (number of patients)	Duration of diabetes in years (number of patients)			
	0-5 (9)	6-10 (12)	11-15 (21)	>15 (8)
6-7 (16)	3	4	8	1
7-10 (24)	5	6	10	3
>10 (10)	1	2	3	4

## Discussion

In the present study, 50 patients with T2DM attending the Diabetic Clinic of the Medical College were studied along with an equal number of age-, sex-, and height-matched controls. Since performing spirometric PFTs is a physically demanding procedure requiring significant coordinated effort, only healthy subjects were included in the study. All of the subjects were non-smokers and without overt cardiac or respiratory illnesses. Subjects who were not able to perform acceptable and reproducible spirometry as per ATS guidelines [14] were excluded from the study.

The mean age of the participants in our study was 56.42 ±10.63 years, which is comparable to that in various similar studies [15,16]. There was no significant difference between the mean age of males (56.89 ±10.64 years) and that of females (55.81 ±10.83 years). When the mean values of spirometric parameters in diabetic patients were compared with controls, there was a significant reduction (p<0.001) in spirometric parameters in diabetic patients. FEF25-75 (73.56 ±29.19% predicted in cases vs. 98.40 ±14.45% predicted in controls with a mean reduction of 24.84) was the most affected followed by PEFR (mean reduction: 15.44), FVC (mean reduction: 12.96), FEV1 (mean reduction: 8.90), and FEV1% (mean reduction: 6.28) in descending order. The average reduction in spirometric parameters was 13.54% as compared to controls.

The degree of derangement in spirometric parameters was also found to be inversely related to the increasing duration of diabetes and increasing levels of HbA1C (a measure of glycemic control). As the duration of diabetes and levels of HbA1C increased, spirometric parameters showed reducing trends. Glycemic control correlated comparatively more strongly with all spirometric parameters than the duration of diabetes. The degree of derangement in spirometric parameters also correlated well with microvascular complications of diabetes. Diabetic patients with microvascular complications of diabetes had a significant decrease in spirometric parameters when compared with those without complications. The degree of derangement was also related inversely to the increasing number of complications. Our observations of mechanical abnormalities in lung functions are consistent with existing literature, demonstrating clear-cut impact in terms of mechanical lung functions in diabetic subjects along with its association with duration, metabolic control, and vascular complications of diabetes.

The main reason for definitive abnormalities of mechanical lung functions observed in the present study could be the comparatively long duration of diabetes (>10 years in 58%) and poor glycemic control (level of HbA1C: >7% in 68%) in the majority of subjects. The mean duration of diabetes was 14.8 years with an SD of 5.8 years; 32% of patients had good glycemic control whereas 68% of patients had poor glycemic control. The mean level of HbA1C was 8.8% with an SD of 2.4%. The majority of patients had an HbA1C level between 7-10% (48%); 20% of patients had an HbA1C level >10%. Various studies that failed to show significant differences in spirometric PFTs between patients with diabetes and normal control subjects, differences with predicted values for the normal population, or a relationship with diabetes control or duration of disease had cited short duration of diabetes or good glycemic control as the main reason for such findings [17,18]. The majority of the subjects in these studies have a duration of diabetes of less than 10 years.

Some of the prospective and cross-sectional studies have shown low vital capacity or restrictive patterns in T2DM [19,20]. A meta-analysis by van den Borst et al. [7] showed a significant association between DM and impaired pulmonary function in a restrictive pattern. These results were irrespective of diabetes duration, smoking, HbA1c levels, and BMI. A study by Davis et al. involving a large number of patients with T2DM showed that VC, FVC, FEV1, and PEFR decreased at an average of between 1.1% and 3.1% of predicted values/year [21]. The study by Ehrlich et al. found that patients with T2DM were at an increased risk of several pulmonary conditions like asthma, COPD, fibrosis, and pneumonia [22]. Other studies have mentioned that no significant differences were observed in patients with T2DM [18-20]. These findings could be attributed to the small sample size in these studies.

The average reduction of 13.54% in spirometric parameters observed in the present study is consistent with a similar level of reduction observed in a community-based cross-sectional study by Davis et al. in 2004 (~10%) [21] and in the Copenhagen City Heart Study (longitudinal analysis of ventilatory capacity) by Lange et al. [5] in 2002 (~8%). The association of the degree of glycemic control with spirometric parameters is also consistent with a similar association demonstrated in a study on the Framingham offspring cohort [23] (2003), a study by Davis et al. [21] (2004), a large prospective longitudinal survey of the pulmonary functions of a cohort of patients with T2DM, and a recent meta-analysis by Díez-Manglano and Asìn Samper [24].

The association of the increasing duration of diabetes with spirometric parameters is consistent with a similar association demonstrated in other studies. Ali et al. [25] demonstrated that diabetic patients with a duration of disease of more than 10 years had a significantly high percentage of PEFR compared to their matching controls, which is in agreement with our results that showed that there were significant differences between diabetic patients and controls in the predicted value of PEFR. The increase in age and longer duration of diabetes were associated with reduced pulmonary functions in the study by Adeyeye et al. [26]. The association of microvascular complications of diabetes with spirometric parameters is consistent with a similar association demonstrated by Baptist et al. [27], Davis et al. [21], and Shah et al. [28].

Simultaneously, there are also various studies, like those by Ozmen et al. [29] and Sinha et al. [18], which have failed to show significant differences in spirometric PFTs between patients with diabetes and normal control subjects, differences with predicted values for the normal population, or a relationship with diabetes control or duration of disease. These inconsistencies may be due to the differences in populations studied in terms of race, age group, smoking history, variations in duration and metabolic control of diabetes, and variations in measurement techniques.

Spirometry is a measure of airflow and lung volumes during forced expiratory maneuvers involving full inspiration. Poor measurement techniques and sub-optimal efforts can provide results that mimic disease patterns. We have avoided these errors by using efficient equipment, which fulfilled the performance and validation criteria as per ATS guidelines [14], and by performing the procedure according to acceptability and reproducibility criteria laid down by ATS guidelines. In addition, a trained technician performed spirometry under direct supervision. 

Among the various spirometric measures, FVC is a measure of lung volume, FEV1% is a measure of patency of large airways, and FEF25-75 is a measure of patency of small airways. In the present study, FEF25-75 was the most affected, and FEV1% was the least affected. FVC was affected intermediately. This pattern of spirometric dysfunction is suggestive of "mixed ventilatory dysfunction". The decreased lung volumes suggest restrictive ventilatory defect and decreased flow rates for small airways suggest early obstructive ventilatory defect.

The proposed hypothesis for lung mechanical dysfunction in diabetes highlights the key role of advanced glycosylated end products (AGEs). There are supposed to be two mechanisms by which AGEs cause lung mechanical dysfunction in diabetes. The first mechanism, i.e., the pro-inflammatory effect of advanced AGEs, suggests obstructive lung mechanical dysfunction in diabetes. The second mechanism, i.e., AGEs-induced functional alterations in the connective tissue of the lung, suggests restrictive lung mechanical dysfunction in diabetes [30]. Hence, the pattern of spirometric dysfunction, i.e., "mixed ventilatory dysfunction", observed in our study is consistent with the proposed mechanisms for lung mechanical dysfunction in diabetes.

Although the pattern of spirometric dysfunction is suggestive of "mixed ventilatory dysfunction", the obstructive disease of small airways is the predominant component [30] as a measure of patency of small airways (FEF25-75) is affected more than the measure of lung volume (FVC). Small airway dysfunction is a feature of early obstructive ventilatory defects.

Although there is significant derangement of spirometric lung functions in patients with T2DM in comparison with controls, this dysfunction is not severe enough to be clinically significant. In other words, it can be stated that diabetic subjects have sub-clinical mechanical pulmonary dysfunction. Consistent with this observation, there have been no reports of functional limitations of activities of daily living attributable to pulmonary disease in patients with diabetes so far.

A potential concern about this study involves the use of spirometry as a primary outcome measure. The most important concern in lung function testing is test quality. Variability is greater in PFT than in most other clinical laboratory tests. However, in the present study, measurements were made by adhering to standardized guidelines of ATS and were in accordance with other similar studies.

## Conclusions

There is significant sub-clinical dysfunction of spirometric lung functions in type 2 diabetic patients. Spirometric lung dysfunction is directly related to glycemic control and the duration of diabetes. The degree of glycemic control is a comparatively strong determinant than the duration of diabetes. Spirometric lung dysfunction also correlates well with microvascular complications of diabetes. Among the various microvascular complications, retinopathy correlates best with various spirometric parameters. The study results highlight the need to include PFTs in the periodic check-up as part of the comprehensive management of diabetic patients. Hence, pulmonary function should be included in the periodic comprehensive diabetic check for the holistic management of these patients.
